# Supplementary material for: Expression of mitochondrial oxidative stress response genes in muscle is associated with mitochondrial respiration, physical performance, and muscle mass in the Study of Muscle, Mobility, and Aging
Source: Aging Cell. 2024 Jun 3;23(6):e14114. doi: 10.1111/acel.14114 (PMC11166362; doi:10.1111/acel.14114)
Supplement: Supplementary file 1 — Table S1. [file ACEL-23-e14114-s001.pdf]

Supplemental Table 1

Gene_symbol	ensembl_gene_id	Cellular localization
CAT	ENSG00000121691	Mitochondria
GPX1	ENSG00000233276	Mitochondria
GPX2	ENSG00000176153	Extra-mitochondria
GPX3	ENSG00000211445	Extra-mitochondria
GPX4	ENSG00000167468	Extra-mitochondria
GPX5	ENSG00000116157	Extra-mitochondria
GPX6	ENSG00000164294	Extra-mitochondria
GRX1	ENSG00000173221	Extra-mitochondria
GRX2	ENSG00000023572	Mitochondria
GRX3	ENSG00000108010	Mitochondria
PRX1	ENSG00000117450	Extra-mitochondria
PRX2	ENSG00000167815	Extra-mitochondria
PRX3	ENSG00000165672	Mitochondria
PRX4	ENSG00000123131	Extra-mitochondria
PRX5	ENSG00000126432	Mitochondria
PRX6	ENSG00000117592	Extra-mitochondria
SOD1	ENSG00000142168	Extra-mitochondria
SOD2	ENSG00000112096	Mitochondria
SOD3	ENSG00000109610	Extra-mitochondria
TRX1	ENSG00000136810	Extra-mitochondria
TRX2	ENSG00000100348	Mitochondria

Supplemental Table 2

Sample	Number of raw pairs	Alignment Rate	Number of aligned reads	Duplication Rate	Number of aligned reads deduplicated
1	96393453	95.67%	184441951	78.70%	39359245
2	100059418	96.13%	192367958	68.10%	61433169
3	93532566	96.08%	179727375	68.50%	56555649
4	90130020	95.93%	172917121	62.90%	64215917
5	93513176	95.74%	179061926	66.50%	60023883
6	93399507	96.03%	179390873	78.60%	38357663
7	91533891	96.04%	175814274	75.50%	43079694
8	95660352	95.15%	182046440	86%	25426123
9	92869196	95.60%	177559973	73.80%	46546318
10	94206508	95.61%	180140177	65.40%	62368154
11	91580510	96.24%	176269851	68.30%	55963786
12	96476311	96.31%	185835269	65.40%	64275793
13	93256753	96.06%	179170646	69.90%	53926180
14	94737694	96.51%	182871302	70.80%	53335997
15	94853873	94.61%	179484404	91.60%	15094298
16	93254346	96.10%	179236781	79.80%	36214863
17	94768451	96.06%	182078220	70.30%	53994168
18	92703785	96.36%	178650402	61.20%	69327789
19	92792742	96.33%	178781101	65.80%	61190811
20	95448973	96.50%	184214920	71.60%	52242426
21	96386549	95.70%	184490763	65.20%	64208390
22	94049712	95.77%	180144045	85.40%	26374250
23	92534259	95.52%	176781852	82%	31751068
24	96136369	95.47%	183567705	77.70%	40894545
25	92684795	96.66%	179169760	79%	37553138
26	94274385	95.62%	180291886	81%	34329087
27	95238852	95.32%	181565967	81.80%	32954237
28	94658476	95.26%	180343006	89.90%	18235077
29	92917699	95.43%	177341522	75.20%	43975639
30	97826542	96.21%	188243115	72.50%	51785757
31	93211235	95.45%	177942884	83.40%	29533846
32	95606463	95.69%	182973610	75.20%	45433035
33	95441961	96.31%	183849801	74.60%	46701458
34	96075319	96.12%	184697195	74.20%	47673981
35	95074060	96.35%	183208134	70.80%	53417857
36	91693188	95.82%	175727627	77.60%	39393394
37	92617017	92.67%	171647448	90.10%	16974404
38	95760744	95.74%	183363658	78%	40363306
39	93959617	94.95%	178419945	82.10%	32008520
40	97687230	95.69%	186945742	72.80%	50891281
41	95290985	96.08%	183118608	76.10%	43755133

42	96405400	96.10%	185288591	71.30%	53153408
43	91618248	95.70%	175361499	74%	45638953
44	90604635	95.59%	173221766	79.70%	35235993
45	91908525	94.24%	173221121	73.90%	45147052
46	93339668	95.45%	178183219	86.60%	23847280
47	93228195	95.38%	177838787	80.10%	35379447
48	94185936	95.70%	180277596	85.90%	25478974
49	93076777	95.60%	177965955	74.90%	44623024
50	92853231	95.51%	177372625	67.50%	57638727
51	95924802	95.83%	183844971	83.20%	30890475
52	91701902	93.61%	171676642	88.50%	19794352
53	93978044	95.60%	179680494	82.20%	31952553
54	93498088	94.84%	177339169	85.90%	25052790
55	91373701	95.10%	173792287	86%	24339052
56	92633161	94.21%	174537705	92.40%	13282764
57	93632029	94.82%	177555337	74.70%	44895907
58	96935810	96.11%	186322556	66.40%	62655363
59	95970326	95.97%	184214134	68.70%	57608266
60	92800167	95.46%	177179393	86.60%	23666468
61	102699034	96.30%	197801041	64.90%	69504546
62	92654946	94.91%	175870882	85%	26296527
63	92637261	94.25%	174620099	85.40%	25547736
64	93933534	94.91%	178301144	86%	24893302
65	94850969	94.69%	179622664	91.10%	15967433
66	93576231	95.41%	178562087	80.90%	34065450
67	90485671	95.26%	172384541	75.90%	41587116
68	93379297	94.19%	175909909	91.20%	15397217
69	96498988	95.91%	185101771	73.10%	49753846
70	71461019	95.40%	136344256	59.30%	55504737
71	93752448	95.60%	179248936	59.20%	73073568
72	86411126	96.23%	166306753	58.30%	69376925
73	83235259	96.26%	160250568	55.50%	71262583
74	81261596	96.36%	156615091	60.10%	62471112
75	97783727	95.85%	187450816	57.10%	80348879
76	83767720	95.75%	160407526	57.60%	68092398
77	86729806	95.31%	165318863	57.60%	70036456
78	110942864	95.51%	211919823	57%	91162871
79	104966827	96.19%	201945250	56%	88772846
80	98253075	96.48%	189598150	65.60%	65198970
81	116927639	96.27%	225129926	56.50%	97959452
82	112176949	96.19%	215797160	58.40%	89813138
83	98534039	95.80%	188796585	53.50%	87847469
84	108954720	95.86%	208892196	53.40%	97416327
85	84097255	95.78%	161090321	57.10%	69129756
86	95236027	95.90%	182663085	61.60%	70206810
87	88597819	95.87%	169884224	55.30%	75955704

88	96328455	96.12%	185180033	57.80%	78177200
89	90461109	96.09%	173855662	56.80%	75088574
90	102870253	95.60%	196695477	57.10%	84377364
91	108655599	96.07%	208762677	55%	93925156
92	106334623	95.39%	202873100	58%	85169516
93	95273543	96.52%	183917484	59.50%	74534598
94	92910303	95.10%	176722146	61.70%	67734224
95	85877520	95.74%	164431755	57.30%	70181106
96	101420521	96.01%	194746668	57.80%	82270980
97	106560498	95.79%	204151071	60.10%	81465013
98	88052217	95.85%	168790927	61.40%	65182021
99	86639281	95.98%	166305148	60.20%	66160365
100	110166267	96.12%	211781602	58.40%	88101366
101	68517291	95.90%	131415364	58.40%	54667509
102	82185872	89.49%	147099825	56.90%	63463056
103	81881951	91.49%	149826924	51.60%	72577249
104	79982310	92.80%	148454721	54.20%	68030223
105	80404905	93.43%	150237431	54.10%	68971717
106	80821283	92.89%	150151720	56.80%	64805299
107	81203942	92.14%	149636180	52.50%	71064306
108	83124572	93.65%	155692925	55%	70035174
109	83732758	91.81%	153750133	56%	67697013
110	85425657	93.21%	159252490	55.20%	71368045
111	83608132	92.59%	154821249	55.10%	69477695
112	80057039	92.61%	148280864	51.40%	72014599
113	80421460	92.78%	149231205	55.70%	66145867
114	79661371	93.39%	148792790	52.40%	70837393
115	78552545	93.67%	147161472	49.60%	74116342
116	80742166	93.70%	151310619	53.80%	69932072
117	81952954	93.45%	153170108	51.40%	74467136
118	84605721	92.48%	156488222	53.50%	72754366
119	83317876	93.43%	155693857	54.60%	70715421
120	84072070	92.31%	155213840	55.10%	69658730
121	78879445	93.85%	148060431	54.30%	67613149
122	81065245	93.13%	150987465	52.90%	71065006
123	80217494	93.74%	150386891	52.60%	71283062
124	78825058	93.19%	146907604	53%	69007099
125	82173370	92.22%	151561681	56%	66722801
126	84325245	91.89%	154967259	55.80%	68529272
127	83005575	92.12%	152925352	52.40%	72862136
128	79764300	92.28%	147216468	54.60%	66881432
129	80033284	93.50%	149658311	53.60%	69425984
130	83094845	92.07%	153013625	56%	67339202
131	81833515	93.24%	152604746	53.20%	71429012
132	81767329	92.75%	151672136	54.50%	69022261
133	86613650	93.83%	162536626	50.90%	79868855

134	84575964	93.04%	157374691	52.80%	74277949
135	84551330	93.20%	157608706	57.30%	67364855
136	79377942	92.20%	146379254	54.60%	66496956
137	82220781	93.55%	153841128	53.80%	71087389
138	84490367	93.66%	158262891	49%	80737589
139	79135730	93.46%	147925972	54.60%	67200337
140	79315800	93.29%	147985482	53.80%	68321650
141	83183251	93.78%	156011569	56.20%	68318618
142	80033861	93.20%	149184350	54.50%	67886689
143	81365287	93.54%	152211890	53.10%	71334270
144	78302039	93.65%	146653385	52%	70451521
145	78250641	93.72%	146677528	50.20%	72978169
146	88982141	93.56%	166509772	51.30%	81144529
147	83465583	93.76%	156512508	52.70%	73991949
148	78062842	93.16%	145453918	54.90%	65605230
149	82988051	93.61%	155366184	54.10%	71328165
150	81862895	93.18%	152566757	53.50%	70874912
151	83357795	92.99%	155033190	53.90%	71547631
152	78541474	92.61%	145477010	55.20%	65238613
153	79658492	93.21%	148502581	54%	68314118
154	83568623	93.12%	155630779	51.10%	76070555
155	82216893	93.36%	153507981	54.80%	69417332
156	80284841	93.01%	149342427	53.80%	69032431
157	81855521	92.63%	151649262	57.70%	64177810
158	83777511	92.15%	154404604	56.40%	67384639
159	76397124	92.84%	141852133	52.10%	67974888
160	76699266	93.64%	143635884	52.50%	68190049
161	81119697	92.98%	150848135	57.30%	64381281
162	80828343	93.88%	151762556	53.70%	70201572
163	81754170	92.54%	151315363	54.40%	68934509
164	77182270	92.88%	143378696	54.50%	65188366
165	83277951	91.93%	153115251	54.80%	69242593
166	81434647	93.24%	151858802	53%	71313059
167	80097377	93.07%	149095520	56%	65587909
168	80351222	92.75%	149049068	55.40%	66477867
169	81065470	92.60%	150136734	56.50%	65376792
170	79732292	92.77%	147932802	51.20%	72190979
171	81364923	92.82%	151041094	54.70%	68468523
172	84217555	92.71%	156156243	53.40%	72719070
173	82659700	91.86%	151855951	58.90%	62427158
174	77764144	92.67%	144122794	54.70%	65326768
175	78458276	93.61%	146894807	53.40%	68434532
176	82281245	92.59%	152375583	54.70%	69096970
177	81723750	93.04%	152071478	53.70%	70467804
178	92966002	93.08%	173061954	53%	81409313
179	108642167	92.90%	201857878	54.50%	91931884

180	110042963	93.11%	204915230	55.40%	91326091
181	80782419	91.74%	148214005	55.40%	66133720
182	84525342	93.26%	157656520	54.20%	72184934
183	83707351	92.93%	155580510	56.70%	67374897
184	81396801	92.67%	150863483	54.20%	69072120
185	79461855	92.93%	147683370	53.60%	68516893
186	82835786	93.45%	154824888	56.10%	68011860
187	80121046	91.52%	146653254	56.20%	64198018
188	80677720	93.08%	150191167	51.70%	72467988
189	73976840	92.95%	137522860	55.50%	61139754
190	84296590	92.68%	156244470	54.30%	71397717
191	79357053	92.05%	146094379	57.40%	62260719
192	83738743	92.78%	155381196	55.10%	69733453
193	81718359	93.00%	151991385	55.70%	67276304
194	82041039	92.73%	152145911	53.70%	70411580
195	80029302	93.27%	149286808	54.60%	67736131
196	80785074	92.28%	149102807	58.20%	62289713
197	83843652	89.95%	150834458	55.60%	66959638
198	83590981	90.54%	151374766	54.40%	69016209
199	83437723	90.56%	151119766	61.30%	58460828
200	83200552	90.82%	151130394	58.30%	63046406
201	85626471	91.26%	156284569	54.70%	70851462
202	85639782	91.15%	156127532	55.30%	69759580
203	82537377	92.16%	152128868	54.80%	68808968
204	85570850	91.07%	155854052	55.50%	69431392
205	85934669	88.37%	151874286	57.70%	64291224
206	82777122	89.44%	148070369	56.30%	64655527
207	84965709	91.77%	155946997	60.90%	60917009
208	83933360	91.66%	153860969	56.30%	67229061
209	84894553	91.66%	155625165	54.60%	70729417
210	86259859	91.23%	157396509	57.40%	67053274
211	84346300	90.21%	152170674	60.30%	60352643
212	81677653	92.44%	151003784	54%	69511530
213	84048781	90.99%	152954587	54.50%	69606002
214	83654135	91.72%	153456971	56.10%	67299283
215	82130420	91.59%	150453661	55.80%	66435733
216	86286063	92.62%	159833996	55.20%	71548012
217	81626143	92.37%	150800166	53.50%	70150436
218	88346127	92.33%	163143773	61.10%	63421969
219	84865918	91.48%	155278413	59.90%	62191416
220	84973197	91.01%	154664003	57.10%	66420352
221	85254795	91.64%	156259490	52.70%	73942216
222	83789654	91.01%	152511134	58.80%	62864169
223	95815599	92.47%	177201844	57.80%	74694621
224	81589169	92.29%	150602813	53.40%	70253955
225	84008407	93.45%	157012490	54.40%	71645670

226	91458190	92.84%	169827777	52%	81521417
227	85816384	92.18%	158208155	51.90%	76103627
228	85124387	92.51%	157502606	53.30%	73491302
229	95718199	92.66%	177393846	50.90%	87012401
230	88765558	91.84%	163040208	50.20%	81131345
231	85307518	92.10%	157139563	47.20%	82950928
232	85215525	92.17%	157078878	52.20%	75159281
233	92867670	92.78%	172320565	50.30%	85711818
234	95802997	92.10%	176464214	51%	86392608
235	90056750	92.42%	166462816	52.40%	79208945
236	88674254	92.26%	163617746	49.10%	83319295
237	87963713	93.34%	164213747	49.40%	83028930
238	92809866	92.70%	172061848	51.70%	83186393
239	87668758	92.36%	161935944	54.80%	73257571
240	83691132	93.13%	155876722	52.30%	74285916
241	87620635	93.43%	163722214	48.50%	84303328
242	87370476	93.83%	163955893	51.50%	79457633
243	85183296	93.51%	159304394	48.90%	81388632
244	85305143	91.00%	155259108	59.40%	62964001
245	87686269	91.76%	160913835	50.10%	80229213
246	85759147	92.88%	159301455	45.80%	86380616
247	86761827	92.46%	160444212	52.30%	76597750
248	95574789	93.14%	178043997	53.60%	82642295
249	84799240	91.03%	154390548	58.90%	63417450
250	92994203	92.25%	171580708	54.90%	77303002
251	90002275	93.51%	168319214	52.50%	79979804
252	93496332	92.41%	172791290	53.20%	80942510
253	93429096	93.13%	174026090	53.40%	81155282
254	92210444	93.87%	173118774	49.60%	87229532
255	86830309	93.01%	161524479	51.20%	78790992
256	85668489	93.69%	160529297	53.20%	75101647
257	82811822	93.50%	154851807	56.70%	67098167
258	92210607	92.75%	171043151	56.20%	74920206
259	86670973	92.98%	161175596	54%	74204742
260	85317863	92.59%	157985436	53.60%	73349237
261	84376356	93.29%	157430753	52%	75551090
262	88846406	93.69%	166474759	52.80%	78535808
263	84250864	93.37%	157323970	49.90%	78810698
264	83884503	93.41%	156707758	55.70%	69362822
265	84039086	93.25%	156727952	58.10%	65613249
266	85755986	93.72%	160745223	52.40%	76444502
267	84323917	93.02%	156873725	55.10%	70465299
268	86565553	93.53%	161926567	50.80%	79604802
269	86567025	93.16%	161289477	50.10%	80523325
270	88494339	92.87%	164361568	52%	78929966
271	86623009	93.02%	161151152	51.20%	78600588

272	86327648	93.10%	160745980	54%	73918147
273	83141346	93.24%	155036034	53.30%	72386235
274	83302444	93.24%	155343331	60.30%	61680278
275	84909067	93.47%	158736296	53.10%	74517144
276	94998934	93.04%	176782669	54.20%	80973713
277	85473106	91.72%	156783969	48%	81529587
278	84419283	93.08%	157151408	49.60%	79147797
279	84339155	92.54%	156093345	54.10%	71659275
280	82898656	93.49%	155005772	57.10%	66469529
281	88618338	92.36%	163687680	55.50%	72864712
282	84028068	92.83%	155998753	55.40%	69596791
283	86880065	92.54%	160795831	54.20%	73623117
284	84845899	93.53%	158711551	51.20%	77485314
285	84860629	92.11%	156330197	52.40%	74379075
286	84257108	93.42%	157421881	52.40%	74920353
287	84280613	93.66%	157878588	50.10%	78725756
288	84861735	93.52%	158721939	53.70%	73496627
289	85386584	93.20%	159158664	51.50%	77183783
290	85547278	91.97%	157357004	64.40%	56086510
291	85506856	92.17%	157623630	50.60%	77883014
292	87465435	92.87%	162465151	48.70%	83288893
293	89476865	93.44%	167220449	50.30%	83141110
294	82110594	93.53%	153591414	55%	69078281
295	84093778	92.59%	155729679	60.40%	61678538
296	83423489	93.01%	155185118	59.60%	62749001
297	85932540	93.29%	160337553	59.40%	65131305
298	83538416	93.34%	155953809	52.40%	74289150
299	84091188	93.52%	157277665	49.20%	79941873
300	90069946	93.01%	167540972	52.10%	80254592
301	84061111	93.66%	157457891	50.80%	77394403
302	83155272	93.16%	154932286	57.50%	65789921
303	83585064	92.55%	154711276	56.30%	67639225
304	84628574	93.46%	158188844	55.90%	69742868
305	83011782	92.14%	152977493	54.20%	70059215
306	81280441	93.78%	152451484	50.80%	74998130
307	84245261	92.76%	156299058	54%	71892451
308	84725802	92.59%	156893654	51.20%	76591677
309	84866329	92.66%	157282114	52.60%	74575212
310	85688224	92.84%	159099875	53.30%	74358069
311	85437828	91.87%	156986962	62.70%	58478149
312	84433153	92.86%	156816220	55.70%	69523283
313	84621049	92.92%	157251935	53.20%	73551058
314	83144014	93.91%	156162315	50.30%	77646238
315	83362769	92.86%	154822328	50.70%	76272766
316	84481479	93.31%	157655895	49.30%	79968258
317	86230336	93.73%	161655502	50.80%	79536622

318	84799431	92.73%	157261019	49.80%	78957526
319	78405835	93.18%	146109354	48%	75928263
320	88892787	93.76%	166692480	50.80%	82020430
321	85070862	93.32%	158769321	48%	82521937
322	84947408	94.30%	160214849	50.80%	78829492
323	92407695	93.73%	173228551	52.50%	82368708
324	84891874	94.27%	160048590	50.70%	78926705
325	85709910	94.39%	161804370	51.50%	78417315
326	83101247	94.01%	156241366	48.50%	80410232
327	82308793	93.72%	154274395	52.10%	73843575
328	87109225	94.00%	163759092	51%	80301894
329	86532923	92.20%	159572561	50.90%	78415267
330	90737882	90.54%	164315402	54.90%	74053890
331	83355319	92.29%	153862208	50.30%	76523033
332	85026830	93.60%	159169119	51.60%	76985647
333	83133743	92.89%	154448461	52.50%	73314153
334	81974796	92.52%	151679531	49.70%	76328624
335	85411349	93.16%	159131845	48.70%	81630732
336	87230169	89.93%	156896035	51.20%	76597809
337	86522843	93.28%	161421146	52.10%	77300193
338	83912636	92.91%	155918473	47.40%	81939343
339	96064117	93.21%	179087734	55.50%	79780353
340	91874480	92.19%	169398960	54.10%	77734137
341	90458184	93.45%	169069924	54.20%	77368105
342	92262874	92.32%	170355177	56.30%	74361329
343	88623713	93.33%	165428691	54.40%	75387321
344	87207805	91.69%	159918728	62.10%	60610291
345	91004676	91.07%	165761485	65.50%	57159741
346	90916275	93.80%	170560323	54.10%	78288436
347	82776921	91.55%	151571920	65.70%	52028736
348	92768155	91.99%	170681073	52.80%	80587319
349	87856604	91.93%	161536091	56%	71121274
350	94246257	93.71%	176642932	54%	81343271
351	90609020	92.28%	167228668	60.30%	66404884
352	96498298	92.76%	179016244	60%	71599729
353	90315456	92.62%	167293252	57.70%	70834030
354	91413142	91.00%	166373604	59.60%	67260982
355	87915585	92.42%	162503027	61.70%	62305118
356	91125683	92.91%	169336075	57.40%	72191861
357	91398473	92.76%	169571520	54.20%	77661393
358	95481994	91.81%	175329119	60.40%	69382289
359	110818214	92.60%	205241457	57.60%	87117111
360	94806068	92.95%	176246636	54.70%	79789029
361	89836893	92.71%	166580194	56.10%	73142972
362	98638711	93.23%	183921845	56.70%	79659069
363	93853377	92.12%	172917042	55.50%	77019448

364	100275484	91.79%	184078267	58.40%	76604986
365	92495429	93.01%	172062371	54.80%	77718704
366	88715899	92.84%	164731494	55.50%	73260860
367	92461693	93.31%	172556715	55.80%	76329898
368	102100512	92.74%	189382528	54%	87061978
369	90504044	92.70%	167798855	60.10%	66899554
370	89709289	93.94%	168552896	52.20%	80537509
371	87126691	93.41%	162764208	60.10%	64993578
372	84451594	93.81%	158447618	54.30%	72379951
373	92484999	93.30%	172582788	56.20%	75514636
374	95069991	92.37%	175627025	53.60%	81522149
375	91417795	93.66%	171251489	54.30%	78191020
376	87960991	90.90%	159916106	59.70%	64483465
377	99886475	93.44%	186660828	57.40%	79514036
378	87890978	92.47%	162553511	57.30%	69367802
379	92017579	93.07%	171275765	61.20%	66437963
380	87881414	93.31%	163996843	54.10%	75296785
381	88237604	93.83%	165581376	52.10%	79392676
382	87808127	93.01%	163340130	55.70%	72401527
383	91892021	94.02%	172792672	52.20%	82574586
384	86824993	93.68%	162683119	53.70%	75385841
385	83169708	92.44%	153768393	64.50%	54605416
386	92957654	91.89%	170829216	61.60%	65562022
387	90627760	93.92%	170231373	63.60%	61998824
388	94159915	93.71%	176478389	53.60%	81955000
389	93895734	92.31%	173357587	67%	57291911
390	93110943	91.98%	171288798	65.50%	59067248
391	91179562	93.39%	170307295	60.70%	66994666
392	94007538	94.13%	176984606	57%	76037137
393	92783466	91.90%	170532129	65.30%	59175784
394	93347423	93.30%	174193583	54.10%	80031876
395	93719900	92.07%	172581611	56.80%	74499816
396	94289838	91.92%	173338731	68.30%	54872025
397	94653775	93.44%	176883243	62.80%	65720019
398	94164370	93.32%	175752605	61.90%	66968050
399	88567436	93.30%	165258210	69.10%	51033967
400	98416526	92.21%	181506660	61%	70857795
401	95476259	92.08%	175822231	74%	45652965
402	92721577	93.59%	173558028	66.90%	57505064
403	92259150	91.10%	168094982	66.80%	55805579
404	88083362	93.76%	165177022	58.10%	69219073
405	92673640	92.23%	170944821	68.20%	54331377
406	91470878	90.71%	165950945	68.30%	52630329
407	92289829	91.24%	168411040	66.50%	56472297
408	88452199	92.23%	163152205	63.10%	60181547
409	94457069	91.78%	173385603	69.20%	53418541

410	90804922	90.60%	164542909	59.40%	66759518
411	92170656	92.31%	170159210	58.80%	70080399
412	88015234	90.63%	159541716	67.40%	52066946
413	90896266	92.02%	167291419	66.50%	55964132
414	95267470	93.90%	178918725	63.10%	66019900
415	90307683	89.91%	162395740	75.20%	40311703
416	89954030	93.51%	168237445	72.70%	45963463
417	92393129	90.77%	167722925	58.60%	69448174
418	96504239	92.38%	178305273	54.50%	81161684
419	94181054	92.24%	173736160	53.60%	80633299
420	88132332	92.81%	163596957	55.20%	73288089
421	92289521	93.42%	172434278	56.30%	75417880
422	93722558	91.93%	172308930	59.10%	70468747
423	91533009	90.67%	165985429	72.30%	45947853
424	98072046	92.20%	180847605	66.80%	59997635
425	94242577	88.97%	167693401	61.30%	64908370
426	90280373	91.21%	164689198	66.60%	54965722
427	93086902	94.13%	175245153	62.80%	65237940
428	88128911	92.82%	163601591	63.40%	59947062
429	93573424	93.78%	175506311	62.50%	65822250
430	93868423	93.42%	175381316	57.60%	74397340
431	98482895	90.88%	179010692	73.80%	46966176
432	93638980	93.02%	174207479	64.10%	62462076
433	89719416	93.27%	167369730	65.70%	57463722
434	95073085	91.95%	174833170	61.70%	66880236
435	89822530	92.97%	167011901	67.30%	54612076
436	91834032	92.67%	170198511	61.20%	66085430
437	92515385	89.52%	165636384	71.80%	46708002
438	81638563	93.65%	152909707	55.20%	68573636
439	100452737	93.09%	187024703	54.60%	84975617
440	96805206	92.74%	179556773	65.90%	61168508
441	98930632	93.51%	185023729	58.70%	76505368
442	96017904	92.77%	178147884	58.20%	74503587
443	90936815	93.73%	170469363	58.90%	70128609
444	89671949	92.11%	165189706	63.80%	59734038
445	93655808	93.56%	175242283	56.20%	76800194
446	92481706	93.39%	172736906	60.80%	67797725
447	92242548	93.29%	172115150	58.20%	71974570
448	98958108	92.46%	183000445	54.90%	82605208
449	89329760	93.54%	167121757	57.40%	71144290
450	92546113	92.53%	171258949	52.70%	80939003
451	95495914	90.44%	172734328	64.70%	60986570
452	90617866	91.86%	166476607	56.70%	72097969
453	85249239	92.77%	158175937	50%	79019520
454	96473089	93.21%	179838851	61.30%	69667130
455	87622388	93.39%	163658186	52.40%	77843675

456	106753538	95.40%	203694475	53.80%	94184243
457	97876344	94.93%	185836410	59.10%	75983609
458	96724752	94.96%	183708439	56.70%	79591673
459	98803703	94.37%	186480153	56.10%	81804328
460	93016321	94.98%	176700272	54%	81212759
461	92084819	94.83%	174656278	55.80%	77212005
462	95001560	93.67%	177982126	57.20%	76218237
463	106809646	94.65%	202187866	57.80%	85413716
464	87839255	94.12%	165355226	52.80%	77967579
465	105811413	94.38%	199739189	56.70%	86405329
466	95541031	91.59%	175007190	69.10%	54148777
467	100430849	94.49%	189789449	58.90%	77935513
468	105982368	93.82%	198873360	56.20%	87132432
469	101514895	94.94%	192751591	50.30%	95763249
470	94660040	89.61%	169654972	59.10%	69446557
471	101901254	95.58%	194798665	54.90%	87802945
472	94699721	94.57%	179109617	57.20%	76588848
473	105237557	94.78%	199487211	54.10%	91466671
474	95567120	96.24%	183956252	62.70%	68536981
475	94395254	96.17%	181562730	58.70%	74947279
476	98761952	94.66%	186975979	58.10%	78341010
477	92467802	94.89%	175485793	59.10%	71696131
478	96982315	94.40%	183094897	61.40%	70687779
479	101831651	94.93%	193331574	53.20%	90575634
480	97300773	95.01%	184887565	52.60%	87728858
481	96666603	93.43%	180640088	63.40%	66139023
482	102251855	95.72%	195760752	58.30%	81682880
483	97637176	94.63%	184781648	52.90%	86991385
484	101283273	93.99%	190390008	57.20%	81456991
485	101091302	94.91%	191881678	57.90%	80701975
486	93013111	94.11%	175065722	59.80%	70354519
487	105604071	94.43%	199445496	54.20%	91328980
488	93988400	93.25%	175281809	64.30%	62488580
489	99689678	94.93%	189267116	59.30%	77096563
490	103505998	95.05%	196756837	58.80%	80978414
491	97155437	94.88%	184359341	61%	71943927
492	97162311	94.64%	183899317	64.20%	65825221
493	97734107	94.45%	184624265	62.60%	69118740
494	97047216	94.80%	183998443	63.20%	67785009
495	97440239	94.48%	184125148	54.40%	84029016
496	94981444	94.62%	179740485	62.30%	67796232
497	90898018	93.98%	170847926	62.10%	64797984
498	98192837	95.43%	187415265	57.50%	79655358
499	94635900	95.09%	179972994	57%	77352751
500	96304744	95.18%	183331357	58.10%	76877869
501	99152855	94.85%	188094606	52.80%	88830603

502	87912768	94.13%	165498166	57.90%	69747262
503	102072863	94.93%	193786918	58.80%	79862675
504	95626828	94.70%	181125533	62.10%	68618652
505	95462159	95.32%	181984022	58.90%	74786308
506	86963075	94.99%	165212291	59.70%	66591725
507	102034874	94.81%	193475179	62.70%	72089086
508	93891414	94.86%	178132723	61.10%	69241995
509	95377432	94.81%	180862233	61.40%	69852609
510	105657361	94.56%	199815540	59.40%	81070642
511	94505366	95.15%	179845898	54.60%	81647583
512	93503827	95.19%	178005941	92.80%	12853785
513	97584136	94.64%	184705043	55.40%	82434795
514	94742267	94.40%	178876936	57.80%	75464728
515	96662924	95.12%	183899328	60.60%	72487446
516	95153939	94.63%	180092578	60.40%	71351472
517	94091898	93.90%	176704699	62.10%	66893054
518	92269025	94.15%	173743946	59.40%	70526796
519	94381366	94.39%	178175304	56.20%	77967554
520	95654141	95.06%	181862423	58%	76350924
521	97093342	94.96%	184396026	58.60%	76254633
522	92388585	95.13%	175771350	58.80%	72358164
523	92060426	94.69%	174350333	58%	73313254
524	109881129	94.29%	207219855	57.20%	88731020
525	101050070	95.68%	193365348	59%	79184265
526	96318962	94.25%	181563940	58.90%	74608502
527	91688583	94.76%	173766923	58.80%	71569344
528	98328743	95.19%	187205952	58.30%	78136105
529	121446366	94.15%	228685610	57.70%	96642845
530	100138016	94.31%	188880646	61.80%	72140808
531	94415088	95.10%	179586762	57.30%	76701379
532	102141157	95.18%	194426179	55.30%	86963987
533	97730240	95.01%	185713182	54.70%	84165544
534	96131064	95.57%	183745808	55.20%	82243616
535	95363612	94.02%	179323841	59%	73469130
536	102903390	94.13%	193719440	61.20%	75199767
537	100346986	92.46%	185567738	62.10%	70414077
538	100722120	94.73%	190831813	57.70%	80788804
539	95881211	94.30%	180836733	60.70%	71070593
540	95567678	95.28%	182111290	52.60%	86301224
541	97359400	95.05%	185084691	59.30%	75373328
542	97578199	94.91%	185213389	61.30%	71606892
543	96910187	95.58%	185246359	53.70%	85782395
544	112780954	95.18%	214694984	52.20%	102724183
545	93275663	95.35%	177880631	56.20%	77840263
546	83354267	94.64%	157767241	50.80%	77638649
547	90439586	95.09%	171994479	59.60%	69448972

548	103168200	93.69%	193320598	52.20%	92492657
549	98614058	95.08%	187530774	52.90%	88392266
550	98772349	94.43%	186545174	51.80%	89907393
551	96029438	91.99%	176675593	58.20%	73793839
552	94987817	94.13%	178823254	55.70%	79172902
553	98177356	95.20%	186920472	52.80%	88148676
554	94374784	94.06%	177536940	56.20%	77690572
555	99184295	94.78%	188008498	61.80%	71824464
556	96475347	93.23%	179896780	56.40%	78377785
557	95325137	94.82%	180772579	55.80%	79913858
558	99134429	96.11%	190559341	62.80%	70917976
559	95543580	94.65%	180869530	57.70%	76457595
560	101595656	95.06%	193147151	55.40%	86101711
561	91435512	94.70%	173172445	55%	77845967
562	95844954	93.06%	178382933	60%	71414095
563	93400224	94.81%	177107821	55.50%	78810835
564	97693345	93.78%	183240742	55.80%	81060064
565	111457841	94.01%	209556407	54.40%	95599806
566	100924314	94.69%	191128298	60.80%	74961152
567	95146106	94.99%	180753766	60%	72328773
568	92308177	94.52%	174506357	60.40%	69065020
569	93055956	94.71%	176261034	53.60%	81708334
570	94736443	94.54%	179120753	59.50%	72515103
571	101645086	94.82%	192751968	59.50%	78029324
572	95245554	93.82%	178711513	58.70%	73752412
573	94462242	94.09%	177753492	58.60%	73591688
574	94920698	94.50%	179406360	57.90%	75451736
575	93789701	95.31%	178776881	57.80%	75530134

Supplemental Table 3

Gene_symbol	ensembl_gene_id	Base Mean	Log 2-Fold Change	Standard Error	Wald Statistic	Unadjusted p-value	FDR adjusted p-value
CAT	ENSG00000121691	3536.640566	0.020799112	0.01104257	1.88353845	0.05962743	0.083478408
GPX1	ENSG00000233276	1606.329124	0.029656353	0.01414079	2.09722027	0.03597408	0.053961121
GPX2	ENSG00000176153	0.92068084	-0.165889668	0.09453044	-1.754881	0.07927967	0.104054566
GPX3	ENSG00000211445	2841.022862	-0.051201476	0.02124657	-2.4098701	0.0159582	0.027926854
GPX4	ENSG00000167468	3526.885745	0.007563184	0.01457408	0.51894748	0.60379737	0.633987236
GPX5	ENSG00000116157	147.9612591	-0.005490288	0.01434978	-0.3826044	0.70201311	0.702013113
GPX6	ENSG00000164294	76.44044373	-0.071198016	0.02335975	-3.0478932	0.00230452	0.004399535
GRX1	ENSG00000173221	1037.515987	-0.07024026	0.02151681	-3.2644365	0.00109682	0.002559247
GRX2	ENSG00000023572	212.0634461	0.081154104	0.01309099	6.19923588	5.67E-10	3.97E-09
GRX3	ENSG00000108010	830.1114115	0.016501173	0.00705916	2.33755493	0.01941035	0.031355178
PRX1	ENSG00000117450	4508.929957	-0.039831525	0.01098233	-3.6268749	2.87E-04	8.61E-04
PRX2	ENSG00000167815	5205.327895	0.08403996	0.01156045	7.2696099	3.61E-13	7.57E-12
PRX3	ENSG00000165672	6278.727427	0.040946481	0.00981352	4.1724552	3.01E-05	1.05E-04
PRX4	ENSG00000123131	383.2419114	-0.047625975	0.01341724	-3.5496115	3.86E-04	0.001012725
PRX5	ENSG00000126432	3652.338857	0.068025145	0.01423265	4.77951467	1.76E-06	7.38E-06
PRX6	ENSG00000117592	8536.515726	0.028092468	0.00902325	3.11334166	0.00184982	0.003884617
SOD1	ENSG00000142168	3355.603106	0.006267947	0.01105577	0.56693895	0.57075566	0.630835204
SOD2	ENSG00000112096	17284.53832	0.08830396	0.01324324	6.66785309	2.60E-11	2.73E-10
SOD3	ENSG00000109610	154.289445	-0.059715478	0.035917	-1.6625966	0.09639322	0.119073977
TRX1	ENSG00000136810	623.353772	-0.012914325	0.01628655	-0.7929442	0.42781034	0.499112063
TRX2	ENSG00000100348	2592.598218	0.060850851	0.01082092	5.62344331	1.87E-08	9.83E-08

Supplemental Table 4

Gene_symbol	ensembl_gene_id	Base Mean	Log 2-Fold Change	Standard Error	Wald Statistic	Unadjusted p-value	FDR adjusted p-value
CAT	ENSG00000121691	3592.269893	0.020388334	0.0165513	1.2318302	0.21801252	0.331462201
GPX1	ENSG00000233276	1646.479136	0.034821591	0.0209871	1.6591916	0.09707718	0.196342976
GPX2	ENSG00000176153	0.905649986	-0.421707114	0.1450658	-2.9070058	0.00364906	0.008514485
GPX3	ENSG00000211445	2853.575891	-0.037429566	0.0319792	-1.170435	0.24182594	0.338556315
GPX4	ENSG00000167468	3605.99008	-0.012158441	0.0215882	-0.5631988	0.57329951	0.668849423
GPX5	ENSG00000116157	150.5454421	0.030594153	0.0217993	1.4034494	0.16048287	0.280845028
GPX6	ENSG00000164294	76.54495587	0.016771646	0.0359288	0.4668027	0.64064108	0.708076981
GRX1	ENSG00000173221	1052.973874	-0.100403095	0.033604	-2.987828	0.00280968	0.0073754
GRX2	ENSG00000023572	214.7983405	0.070105428	0.0206891	3.3885232	7.03E-04	0.002951344
GRX3	ENSG00000108010	838.7854228	0.017615545	0.0107991	1.6312086	0.10284632	0.196342976
PRX1	ENSG00000117450	4591.513808	-0.069009194	0.0165704	-4.1646142	3.12E-05	2.91E-04
PRX2	ENSG00000167815	5329.397319	0.074544418	0.0181895	4.0982201	4.16E-05	2.91E-04
PRX3	ENSG00000165672	6378.991017	0.076555706	0.0145009	5.2793887	1.30E-07	2.72E-06
PRX4	ENSG00000123131	389.6228736	-0.018189237	0.0205381	-0.8856353	0.37581411	0.493256013
PRX5	ENSG00000126432	3742.08287	0.066436822	0.0216872	3.0634087	0.00218831	0.006564928
PRX6	ENSG00000117592	8688.82927	0.005530201	0.0139186	0.3973245	0.69112821	0.725684624
SOD1	ENSG00000142168	3417.991118	-0.011146816	0.0167137	-0.6669256	0.50481965	0.62360074
SOD2	ENSG00000112096	17516.63393	0.081735281	0.0204854	3.9899299	6.61E-05	3.47E-04
SOD3	ENSG00000109610	150.8857732	-0.003270022	0.0510508	-0.0640543	0.94892697	0.948926967
TRX1	ENSG00000136810	630.5753855	-0.030223607	0.0246937	-1.2239402	0.2209748	0.331462201
TRX2	ENSG00000100348	2650.274681	0.054658119	0.0168583	3.2422103	0.00118606	0.004151226

Supplemental Table 5

Gene_symbol	ensembl_gene_id	Base Mean	Log 2-Fold Change	Standard Error	Wald Statistic	Unadjusted p-value	FDR adjusted p-value
CAT	ENSG00000121691	3585.390585	0.010189612	0.0113074	0.901147	0.3675101	0.615558922
GPX1	ENSG00000233276	1635.574598	-0.016821335	0.0140978	-1.1931929	0.2327938	0.464367537
GPX2	ENSG00000176153	0.920250442	-0.10843793	0.0929261	-1.1669263	0.2432401	0.464367537
GPX3	ENSG00000211445	2864.281901	-0.075568556	0.021428	-3.5266198	4.21E-04	0.004419457
GPX4	ENSG00000167468	3589.335283	-0.004412526	0.0144678	-0.3049889	0.7603746	0.887103668
GPX5	ENSG00000116157	149.6814679	0.012769492	0.014578	0.8759444	0.3810603	0.615558922
GPX6	ENSG00000164294	76.71538891	-0.033008035	0.0238003	-1.3868757	0.1654797	0.406290641
GRX1	ENSG00000173221	1047.642848	-0.052082612	0.0222875	-2.3368576	0.0194466	0.06806306
GRX2	ENSG00000023572	213.1942236	8.68E-04	0.0139702	0.062138	0.9504529	0.997975568
GRX3	ENSG00000108010	834.8093103	0.003685894	0.0071914	0.5125403	0.6082729	0.798358176
PRX1	ENSG00000117450	4578.206837	-0.051338745	0.0108619	-4.7265132	2.28E-06	4.80E-05
PRX2	ENSG00000167815	5286.351421	0.001869263	0.0122899	0.1520974	0.8791101	0.971648035
PRX3	ENSG00000165672	6327.809818	0.03145825	0.0101224	3.1077866	0.0018849	0.009895942
PRX4	ENSG00000123131	388.0849099	0.008715982	0.0138445	0.6295623	0.528981	0.740573403
PRX5	ENSG00000126432	3713.455874	0.005632956	0.0145095	0.3882245	0.6978499	0.862049876
PRX6	ENSG00000117592	8636.675112	-0.005852851	0.0092106	-0.6354496	0.5251353	0.740573403
SOD1	ENSG00000142168	3401.027127	-0.021963822	0.010992	-1.9981722	0.045698	0.137093988
SOD2	ENSG00000112096	17352.42193	0.018829422	0.0138546	1.3590695	0.1741246	0.406290641
SOD3	ENSG00000109610	155.8195125	-0.112999695	0.0356863	-3.1664728	0.001543	0.009895942
TRX1	ENSG00000136810	629.0138476	-0.041550626	0.0163342	-2.5437756	0.0109662	0.046057839
TRX2	ENSG00000100348	2629.257654	-9.63E-06	0.0112872	-8.53E-04	0.9993192	0.999319217

Supplemental Table 6

Gene_symbol	ensembl_gene_id	Base Mean	Log 2-Fold Change	Standard Error	Wald Statistic	Unadjusted p-value	FDR adjusted p-value
CAT	ENSG00000121691	3571.291718	0.011211287	0.0138526	0.8093286	0.4183262	0.627489228
GPX1	ENSG00000233276	1638.93964	0.009448744	0.017391	0.5433136	0.5869139	0.821521591
GPX2	ENSG00000176153	0.914530847	-0.056704787	0.1163233	-0.487476	0.6259212	0.821521591
GPX3	ENSG00000211445	2851.242963	-0.033795366	0.0263004	-1.284976	0.1988007	0.417481539
GPX4	ENSG00000167468	3598.765541	-0.001204347	0.0177946	-0.06768	0.9460401	0.993342067
GPX5	ENSG00000116157	149.5434931	0.033227187	0.0174603	1.9030088	0.0570394	0.19703381
GPX6	ENSG00000164294	76.45041	-0.006446824	0.0293871	-0.219376	0.8263574	0.913342381
GRX1	ENSG00000173221	1042.001154	0.026690029	0.0272123	0.980808	0.3266874	0.527725843
GRX2	ENSG00000023572	213.113651	0.03830537	0.0169269	2.2629841	0.0236367	0.101144012
GRX3	ENSG00000108010	834.6912074	0.014764507	0.0086455	1.7077726	0.0876785	0.230156184
PRX1	ENSG00000117450	4567.218891	-0.054772574	0.0133599	-4.099765	4.14E-05	8.68E-04
PRX2	ENSG00000167815	5291.612745	0.016514817	0.0151116	1.0928554	0.2744573	0.480300288
PRX3	ENSG00000165672	6332.472884	0.028042082	0.012431	2.25582	0.0240819	0.101144012
PRX4	ENSG00000123131	388.0841437	0.01988785	0.0168595	1.179626	0.238149	0.454648079
PRX5	ENSG00000126432	3717.657427	0.032855621	0.0178503	1.8406154	0.0656779	0.19703381
PRX6	ENSG00000117592	8618.505492	-0.014643164	0.0112878	-1.297253	0.194544	0.417481539
SOD1	ENSG00000142168	3393.928407	-0.003649322	0.0134542	-0.271239	0.7862069	0.913342381
SOD2	ENSG00000112096	17357.4281	0.040939991	0.0168627	2.4278361	0.0151892	0.101144012
SOD3	ENSG00000109610	155.5398227	-0.155010779	0.0438706	-3.533363	4.10E-04	0.004308239
TRX1	ENSG00000136810	627.2693115	-1.45E-04	0.02002	-0.007233	0.9942293	0.994229312
TRX2	ENSG00000100348	2632.277607	0.004273069	0.0138585	0.3083347	0.7578276	0.913342381

Supplemental Table 7

Gene_symbol	ensembl_gene_id	Base Mean	Log 2-Fold Change	Standard Error	Wald Statistic	Unadjusted p-value	FDR adjusted p-value
CAT	ENSG00000121691	3591.448278	-0.016333568	0.02539747	-0.643118	0.52014757	0.606838826
GPX1	ENSG00000233276	1635.286662	0.011275682	0.031605	0.35676895	0.72126478	0.75732802
GPX2	ENSG00000176153	0.923960722	-0.368989151	0.21121477	-1.7469855	0.08063985	0.182093355
GPX3	ENSG00000211445	2874.879088	-0.045383919	0.04805962	-0.9443253	0.34500344	0.517505167
GPX4	ENSG00000167468	3589.282415	-0.024173591	0.03239402	-0.7462362	0.45552472	0.562707001
GPX5	ENSG00000116157	149.6223278	6.48E-04	0.03263767	0.01985046	0.98416267	0.984162668
GPX6	ENSG00000164294	76.89552489	0.08815068	0.05285874	1.66766507	0.09538223	0.182093355
GRX1	ENSG00000173221	1047.924975	-0.024251191	0.04914783	-0.4934336	0.62170625	0.687149013
GRX2	ENSG00000023572	213.3906652	0.127321044	0.03054245	4.16865785	3.06E-05	3.22E-04
GRX3	ENSG00000108010	834.384473	0.026404406	0.01566926	1.68510889	0.09196756	0.182093355
PRX1	ENSG00000117450	4579.187885	-0.128075093	0.02403429	-5.3288489	9.88E-08	2.08E-06
PRX2	ENSG00000167815	5294.492168	0.049649165	0.02731575	1.81760194	0.06912499	0.182093355
PRX3	ENSG00000165672	6330.557468	0.068630913	0.02250904	3.0490377	0.00229576	0.012052723
PRX4	ENSG00000123131	387.7326614	0.023251376	0.03015147	0.77115221	0.44061672	0.562707001
PRX5	ENSG00000126432	3713.956045	0.055678424	0.03232615	1.7223956	0.08499788	0.182093355
PRX6	ENSG00000117592	8663.968863	-0.024263245	0.020413	-1.1886173	0.2345903	0.410533027
SOD1	ENSG00000142168	3400.837399	-0.048465734	0.02422776	-2.0004215	0.04545477	0.159091696
SOD2	ENSG00000112096	17371.52306	0.121637594	0.03061064	3.97370249	7.08E-05	4.95E-04
SOD3	ENSG00000109610	156.8308598	-0.179850569	0.08014791	-2.2439833	0.02483348	0.104300606
TRX1	ENSG00000136810	628.6504202	-0.030828476	0.03593097	-0.8579916	0.39089708	0.547255912
TRX2	ENSG00000100348	2633.3436	0.025874756	0.02501905	1.03420204	0.30104172	0.486298162

Supplemental Table 8

Gene_symbol	ensembl_gene_id	Base Mean	Log 2-Fold Change	Standard Error	Wald Statistic	Unadjusted p-value	FDR adjusted p-value
CAT	ENSG00000121691	3557.926407	-0.030349068	0.015417624	-1.96846597	0.049014448	0.17446875
GPX1	ENSG00000233276	1628.663082	0.019402342	0.019293055	1.005664569	0.314576952	0.508162769
GPX2	ENSG00000176153	0.915597597	0.349529335	0.124353879	2.810763432	0.004942411	0.035261374
GPX3	ENSG00000211445	2865.490425	0.040180434	0.02967695	1.353927333	0.175759562	0.369095081
GPX4	ENSG00000167468	3579.788156	0.020945854	0.019938515	1.050522298	0.293478045	0.508162769
GPX5	ENSG00000116157	148.6481679	0.012547368	0.019947818	0.629009554	0.529342802	0.653894049
GPX6	ENSG00000164294	76.22789715	0.087978097	0.03245723	2.710585543	0.006716452	0.035261374
GRX1	ENSG00000173221	1041.145766	0.046023684	0.030569217	1.505556518	0.132181058	0.308422468
GRX2	ENSG00000023572	211.548143	0.070257016	0.018477007	3.802402404	1.43E-04	0.003009294
GRX3	ENSG00000108010	828.9073641	0.019206444	0.00979289	1.961264172	0.049848214	0.17446875
PRX1	ENSG00000117450	4549.437954	-0.012668234	0.015280099	-0.82906758	0.407066165	0.610599248
PRX2	ENSG00000167815	5271.862115	-8.83E-04	0.01672705	-0.05280418	0.957887929	0.957887929
PRX3	ENSG00000165672	6282.480784	0.01692661	0.014036435	1.205905168	0.227854058	0.43499411
PRX4	ENSG00000123131	385.6266566	0.035039922	0.01883315	1.86054489	0.062808473	0.18842542
PRX5	ENSG00000126432	3702.216113	0.012815591	0.019996753	0.640883625	0.521598295	0.653894049
PRX6	ENSG00000117592	8586.790786	-0.004999731	0.012627298	-0.39594625	0.692144678	0.765002012
SOD1	ENSG00000142168	3382.217714	0.003149757	0.015199144	0.207232498	0.835828288	0.877619703
SOD2	ENSG00000112096	17215.38143	0.009041537	0.019004453	0.475758883	0.634246166	0.73995386
SOD3	ENSG00000109610	156.0650668	-0.086321405	0.049453829	-1.74549485	0.080898768	0.212359267
TRX1	ENSG00000136810	623.8251393	0.060872991	0.022349346	2.723703491	0.006455443	0.035261374
TRX2	ENSG00000100348	2618.571945	-0.010610119	0.01546764	-0.68595593	0.492740902	0.653894049
