# Supplementary material for: Expression of mitochondrial oxidative stress response genes in muscle is associated with mitochondrial respiration, physical performance, and muscle mass in the Study of Muscle, Mobility, and Aging
Source: Aging Cell. 2024 Jun 3;23(6):e14114. doi: 10.1111/acel.14114 (PMC11166362; doi:10.1111/acel.14114)
Supplement: Supplementary file 2 — Captions. [file ACEL-23-e14114-s002.docx]

**Table S1.** List of twenty-one oxidative stress response genes, ENSG identifiers, and preferred cellular localizations.

**Table S2.** RNAseq quality metrics for each sample, including: number of raw pairs, alignment rate, number of aligned reads, duplication rate, and number of aligned reads deduplicated.

**Tables S3–S8.** Listings of Ensembl gene (ENSG) associations identified by negative binomial regression for each trait (one table per trait: Max OXPHOS, VO_2_ peak, 400-meter Walk Speed, Leg Strength, Thigh Muscle Mass, and Whole Body D3Cr).
